# An Exploratory Preclinical In Vivo Pilot Study Evaluating the Hair Growth–Promoting Effects of *Rosmarinus officinalis* and *Ricinus communis* Combination

**DOI:** 10.1155/bmri/5037275

**Published:** 2026-05-11

**Authors:** Alex Mathenge, Elizabeth Odongo, Ruth Mauti, Fednard Osee

**Affiliations:** ^1^ Department of Preclinicals and Pharmacognosy, Kabarak University, Nakuru City, Kenya, kabarak.ac.ke

**Keywords:** alopecia, hair growth, *Ricinus communis*, *Rosmarinus officinalis*, traditional medicine

## Abstract

**Background:**

Hair loss is a common condition with significant psychosocial impact. Although pharmacological treatments such as minoxidil are available, their long‐term use may be associated with adverse effects. Plant‐derived products are widely used in traditional medicine for hair care; however, scientific validation of their efficacy remains limited.

**Objective:**

This study is aimed at evaluating the hair growth–promoting effects of a combination of *Rosmarinus officinalis* and *Ricinus communis* oils in a murine model.

**Methodology:**

Essential oil from *R*. *officinalis* was obtained by hydrodistillation, whereas fixed oil from *R*. *communis* seeds was extracted using Soxhlet extraction with hexane. Fifteen mice (*n* = 3 per group) were divided into five groups: rosemary oil, castor oil, combination (1 : 1), 2% minoxidil (positive control), and untreated (negative control). Treatments were applied topically once daily for 28 days. Hair growth was evaluated using hair length measurement, macroscopic observation, and standardized hair regrowth scoring. Data were analyzed using one‐way ANOVA followed by Tukey′s post hoc test. Statistical outputs are presented descriptively due to the small sample size and *p* values are reported for trend identification rather than inferential significance.

**Results:**

The combination group showed higher mean hair length (12.88 ± 0.79 mm) compared with *R*. *officinalis* (12.68 ± 0.25 mm) and *R*. *communis* (10.10 ± 0.22 mm) and showed similar trends to 2% minoxidil (13.06 ± 0.21 mm). One‐way ANOVA showed overall group differences. Statistical outputs are presented descriptively due to the small sample size; *p* values are reported for trend identification rather than inferential significance.

**Conclusion:**

The findings suggest that the combination of *R*. *officinalis* and *R*. *communis* oils may promote hair growth in a murine model. The present findings are derived from a murine model and cannot be directly extrapolated to human use.

## 1. Introduction

Hair holds profound cultural and social significance, serving as a symbol of identity, status, religion, and self‐expression [1]. Beyond its cultural relevance, hair plays an important role in physical appearance and can influence perceptions of attractiveness, health, and personality [2]. From a physiological perspective, hair contributes to protection against environmental factors, assists in thermoregulation, and may serve as an indicator of systemic health. Additionally, hair can retain trace elements, making it useful in forensic and toxicological investigations [3].

Hair loss is a common condition that may result from disruptions in normal hair follicle cycling, structural abnormalities, or external factors such as chemotherapy and radiation therapy [4]. It often has significant psychological consequences, including reduced self‐esteem and increased anxiety [5]. Alopecia refers to hair loss occurring on the scalp or other parts of the body and can be broadly classified into scarring and nonscarring types [6]. Among these, alopecia areata is an autoimmune disorder characterized by patchy hair loss, whereas androgenetic alopecia is a hormonally influenced condition resulting in progressive hair thinning in both men and women [7].

Currently, only a limited number of pharmacological treatments are approved for hair loss, including topical minoxidil and oral finasteride. Although these therapies have demonstrated efficacy, they are associated with adverse effects. For example, minoxidil has been reported to cause facial hypertrichosis and contact dermatitis in some patients, whereas finasteride has been linked to sexual dysfunction, mood disturbances, and postfinasteride syndrome [8]. Moreover, these treatments often require long‐term or continuous use to maintain therapeutic effects

As a result, there has been growing interest in alternative approaches, particularly plant‐based therapies derived from traditional medicine systems such as Ayurveda, traditional Chinese medicine, and African ethnomedicine. These systems utilize a variety of botanical preparations to promote hair growth, improve scalp health, and manage hair loss conditions [9]. Among commonly used plants, *Rosmarinus officinalis* has been traditionally employed to enhance hair vitality and may exert biological effects through antioxidant and potential anti‐androgenic mechanisms [10, 11]. Similarly, *Ricinus communis* oil is widely used for its emollient and conditioning properties, largely attributed to its high content of ricinoleic acid and fatty acids, which may support scalp health [12]. These oils are also frequently incorporated into commercial hair care formulations due to their perceived benefits [13].

Despite their widespread traditional and commercial use, there remains limited scientific evidence evaluating the combined effects of these plant‐derived oils on hair growth. Furthermore, existing studies often focus on individual plant extracts rather than potential synergistic interactions. Therefore, there is a need for systematic preclinical investigations to assess their efficacy using controlled experimental models.

Although individual effects of these oils have been explored, their combined pharmacodynamic interaction remains poorly characterized. The present study was designed as a preliminary investigation to evaluate the hair growth–promoting effects of a combination of *R*. *officinalis* and *R*. *communis* oils using a murine model. This work is aimed at providing foundational data that may inform future studies, including larger preclinical investigations and clinical trials.

## 2. Materials and Methods

### 2.1. Collection and Preparation of Plant Material

Fresh leaves of *R*. *officinalis* and seeds of mature *R*. *communis* were collected from the Kabarak University botanical garden, Nakuru County. The plant materials were authenticated by a qualified taxonomist at Kabarak University, and voucher specimens (AMF 001) were deposited in the institutional herbarium for future reference. The plant material was air dried, then ground into powder and stored separately in airtight containers at room temperature [14, 15].

#### 2.1.1. Preparation of Plant Extracts

*R*. *officinalis* leaf powder was subjected to hydrodistillation with the Clevenger under optimal operational conditions with a temperature of 80°C as described by Elyemni et al. [16]. Two hundred grams (200 g) of *R*. *officinalis* leaf powder was mixed with 1000 mL of distilled water. The distillation process was performed for 3 h and the obtained essential oil was collected [15].

A total of 100 g of the pulverized *R*. *communis* seeds were kept in a thimble holder before placing them into the Soxhlet apparatus. Hexane (300 mL) was dispensed in a round‐bottom flask to initiate the extraction process. The extraction was carried out for 6 h at 60°C. When the solution in the thimble was clear, it signified that the oil was completely extracted from the raw seed and the apparatus was switched off. The collected solvent and the oil were oven dried, cooled in the desiccators, and stored in an airtight container at room temperature. Extraction  yield  (*%*) = (weight  of  extracted  oil/weight  of  plant  material) × 100 (Mohammed et al. 2017).

### 2.2. Preparation of the Test Animals and Sample Size

Adult male and female BALB/c mice (20–25 g) were used. The mice were provided with a chow diet with access to fresh water. The mice were maintained free of specific pathogens at a controlled temperature (23^°^C ± 2^°^C), humidity (50*%* ± 20*%*), and light (12‐h light–dark cycle). A skin irritation test was performed, whereby the hair from the neck and dorsal skin of the mice was shaved, and the area was cleaned with surgical spirit. The extracted oils and the formulation were then applied, and the mice were observed for erythema and edema for a period of 24, 48, and 72 h [18].

Pilot studies recommend a sample size of 3–5 for feasibility. Similar preliminary in vivo pharmacognosy studies commonly employ small sample sizes as exploratory screening tools to identify biological activity prior to large‐scale validation studies. This approach is widely applied in early‐stage medicinal plant research and preclinical drug discovery, where pilot animal experiments are used to assess feasibility and generate initial efficacy signals before powered studies are conducted [19–21].

Results with *n* = 3 are presented as descriptive trends only.

### 2.3. Evaluation of Hair Growth Promoting Effects

Fifteen mice were separated into five groups (*n* = 3/group) and left for 1 week of adaptation. The animals were randomly assigned to treatment groups. Twenty‐four hours before the start of the experiment, the skin surface of the back of the mice was shaved using a shaver. Different treatments were applied to the groups. The first group was applied with *R*. *officinalis* oil topically, the second group was applied with *R*. *communis* oil topically, and the third group was applied with a mixture of *R*. *officinalis* and *R*. *communis* oil. The fourth group received 2% minoxidil (positive control), whereas the fifth group received no treatment and served as the negative control. [22].

A fixed volume of 0.1 mL of each formulation was applied topically to a shaved dorsal area (~2 cm^2^) once daily for 28 days. The combination group received a 1:1 (*v*/*v*) mixture of *R*. *officinalis* and *R*. *communis* oils.

The degree of hair growth was observed by macroscopic assessment with the naked eye; the area of interest was visualized and photographed at specific time intervals (Days 1,7,14, 21, and 28), in order to record the start of the hair regrowth period and the pattern of hair regrowth compared with controls [23].

Hair regrowth scores were used for further assessment; for instance, ranges from 0 to 5: 0 = no hair growth, 1 = less than 20*%* of hair growth, 2 = 20*%*–39*%* of hair growth, 3 = 40*%*–59*%* of hair regrowth, 4 = 60*%*–79*%* of hair regrowth, and 5 = 80*%*–100*%* of hair regrowth as described by Matsuda et al. [24]. A descriptive scale was also used which considered Type IV (high hair density, full, and thick fur), Type III (moderate hair density with no visible skin area), Type II (low hair density with the visualization of the skin), and Type I (uneven hair growth on the test area and skin easily seen) [22].

Hair length was measured by randomly plucking 10 strands from the treated area and measuring using a digital caliper. The length of the hairs of the animals was measured and compared with the length of the hairs from the mice in the control group [25].

### 2.4. Data Monitoring, Presentation, and Analysis

In order to augment the validity of the findings, all laboratory instrumentation was subjected to rigorous calibration and a high‐definition imaging system employed. The animals were maintained under strictly controlled husbandry conditions to safeguard their normal physiological integrity. Hair‐growth parameters and related observations were systematically recorded each week to monitor experimental progress and substantiate the precision of the measurements. [22].

Animals were randomly assigned to treatment groups using a simple randomization procedure. Hair growth assessment was performed by an independent observer blinded to treatment allocation to minimize observational bias.

Data presentation was in the form of photographs taken with a good quality camera to clearly and sequentially show the hair regrowth pattern on the animals. Data tabulated include the length of the hair on the different groups of mice before and after treatment with the various extracts [22].

Data were analyzed using one‐way analysis of variance (ANOVA), followed by Tukey′s post hoc test for multiple group comparisons [26]. Given the limited sample size (*n* = 3 per group), the analysis was considered exploratory in nature, and all statistical outputs were interpreted descriptively to identify potential trends rather than to draw confirmatory conclusions. One‐way ANOVA was employed as an overall global test to evaluate whether observable variability among treatment groups exceeded within‐group variation. Where appropriate, Tukey′s post hoc comparisons were conducted to describe relative differences between groups. However, all inferential statistics, including *p* values, are reported strictly as exploratory indicators of possible effects and should not be interpreted as evidence of statistical significance.

### 2.5. Ethical Consideration

Ethics review was sought from Kabarak University Research Ethics Committee (KUREC‐080524).

Permission to collect data was obtained from the National Commission for Science, Technology and Innovation (NACOSTI) (Research License No. NACOSTI/P/24/39919). All procedures involving the mice were designed to minimize harm and suffering. The test protocol was noninvasive, and a skin irritation test was performed first to ensure the animals were not exposed to toxicity. The experiment was done under the supervision of licensed and experienced laboratory staff, and ethical approval for the study was sought prior. Proper record keeping was also employed as recommended in the Prevention of Cruelty to Animals Act, Act No. 12 of 2012. After the experiments, the animals were gently euthanized and safely disposed of via incineration. Efforts were made to minimize any potential harm or discomfort to the mice involved in the study.

## 3. Results

The yield of *R*. *officinalis* essential oil was 2.3% (*w*/*w*), whereas *R*. *communis* oil was 27%. The individual *R*. *officinalis* oil*, R*. *communis* oil, and the combined *R*. *officinalis*–*R*. *communis* oils experimental groups exhibited a pronounced, progressive increase in hair growth beginning on Day 14 of treatment, consistent with the onset of the anagen phase of the hair cycle. The group treated with the rosemary–castor oil formulation, in particular, showed the highest hair density, as reflected by its superior hair regrowth scores.

One‐way ANOVA suggested differences among the groups (*p* < 0.0001, exploratory). Tukey′s post hoc analysis showed that the combination treatment, and 2% minoxidil group exhibited greater hair growth compared with the negative control (*p* < 0.001). The mean hair length, regrowth scores, descriptive scale, and exploratory *p* values for each group compared with the negative control are presented in Table 1. The *R*. *officinalis* group also showed an increase (*p* < 0.001), whereas *R*. *communis* did not differ from the control (*p* > 0.05). No clear difference was observed between the combination group and the minoxidil group (*p* > 0.05). Pairwise comparisons between all treatment groups with exploratory interpretations are shown in Table 2.

**Table 1:** Hair regrowth parameters in mice after 28 days of topical treatment.

Mice group	Average length of the hair strands (*m* *e* *a* *n* ± *S* *D*[mm])	Hair regrowth scores	Descriptive scale	Comparison versus negative control	*p* value (exploratory)
*Rosmarinus officinalis*	12.68 ± 0.25	4	Type III	Increased hair growth	(*p* < 0.001)
*Ricinus communis*	10.10 ± 0.22	4	Type III	Comparable with control	No clear difference (*p* < 0.998)
*R*. *officinalis*–*R*. *communis* combination	12.88 ± 0.79	5	Type IV	Markedly increased	(*p* < 0.001)
2% minoxidil (positive control)	13.06 ± 0.21	5	Type IV	Markedly increased	(*p* < 0.001)
Negative control	10.58 ± 0.38	3	Type II	Baseline	_

**Table 2:** Pairwise comparisons of mean hair length between treatment groups with exploratory interpretation.

Comparison	*p* value	Exploratory interpretation
Combination versus negative control	< 0.001	Suggestive of difference
Minoxidil versus negative control	< 0.001	Suggestive of difference
*Rosmarinus officinalis* versus negative control	< 0.001	Suggestive of difference
*Ricinus communis* versus negative control	0.60	No clear difference
*R*. *officinalis* versus *R*. *communis*	< 0.001	Suggestive of difference
*R*. *officinalis* versus minoxidil	0.78	No clear difference
Combination versus minoxidil	0.97	No clear difference

*Note:* Due to the small sample size (*n* = 3 per group), *p* values are exploratory and should be interpreted cautiously. All comparisons were performed using Tukey′s honest significant difference (HSD) test following one‐way ANOVA.

**Figure 1:**
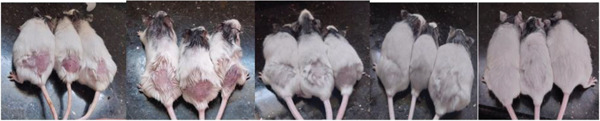
*Rosmarinus officinalis* group on Days 1, 7, 14, 21, and 28.

**Figure 2:**
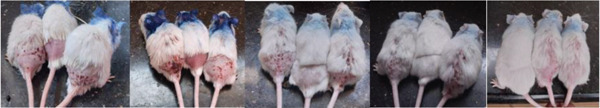
*Ricinus communis* group on Days 1, 7, 14, 21, and 28.

**Figure 3:**
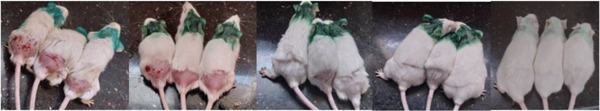
*Ricinus communis*–*Rosmarinus officinalis* combination group on Days 1, 7, 14, 21, and 28.

Hair regrowth progressed across all treatment groups over the study period, with the combination treatment demonstrating enhanced effects compared with individual extracts. Representative macroscopic images of dorsal hair regrowth over 28 days are presented for the *R*. *officinalis* group (Figure 1), *R*. *communis* group (Figure 2), and combination treatment group (Figure 3).

## 4. Discussion

In this study, topical application of *R*. *officinalis* and *R*. *communis* oils demonstrated hair growth–promoting activity in a murine model. The observed effects were consistent with previous findings suggesting that rosemary oil may support hair growth under experimental conditions [8]. Notably, the combination of both oils showed higher hair growth activity compared with the individual treatments, suggesting a possible additive or synergistic effect under the conditions of this study.

The biological activity of *R*. *officinalis* may be attributed to its phytochemical constituents, including 12‐methoxycarnosic acid and carnosic acid. These compounds have been reported to exhibit antioxidant and potential antiandrogenic properties. It has been suggested that rosemary‐derived compounds may influence hair growth pathways by inhibiting 5*α*‐reductase, the enzyme responsible for converting testosterone into dihydrotestosterone (DHT), which is associated with hair follicle miniaturization [27]. In addition, antioxidant activity may reduce oxidative stress in hair follicles, which is a known contributing factor in hair loss. Previous experimental evidence has also indicated that rosemary oil may enhance peripheral blood circulation, which could support nutrient delivery to hair follicles [11, 28].

Mild skin irritation was observed in the group treated with *R*. *officinalis*. This finding is consistent with earlier reports indicating that rosemary oil may cause localized irritation in sensitive individuals [29]. Such reactions have been associated with naturally occurring constituents such as limonene, linalool, and carnosol, which may act as contact allergens in susceptible subjects [30]. These observations suggest that although rosemary oil may have biological activity relevant to hair growth, its potential for irritation should be considered in topical applications.

In contrast, *R*. *communis* oil demonstrated relatively lower hair growth activity compared with rosemary oil and the combination treatment. This may be related to its physicochemical properties, particularly its high viscosity, which may limit dermal penetration and reduce interaction with deeper follicular structures [31, 32]. However, castor oil may still contribute to hair health through its emollient and conditioning effects. Its major constituent, ricinoleic acid, has been reported to support scalp hydration and may reduce hair breakage by improving hair fiber flexibility. Additionally, omega fatty acids present in castor oil have been associated with improved hair quality and density in some nutritional studies [13, 33]. Importantly, castor oil is generally considered nonirritating and has a favorable safety profile for topical use [34, 35].

The combination of rosemary and castor oils produced higher hair growth activity compared with either oil alone and was associated with reduced incidence of irritation. This may be due to dilution of rosemary oil within the castor oil matrix, potentially reducing the concentration of irritant constituents while maintaining bioactivity. Additionally, the presence of castor oil may have improved the spreadability and dermal delivery of rosemary constituents by reducing viscosity and enhancing topical distribution. The absence of irritation in the combination group suggests that formulation effects may influence both efficacy and tolerability.

It is worth noting that this study was conducted under controlled experimental conditions using a small sample size. Therefore, the findings should be interpreted as preliminary evidence of biological activity rather than confirmatory results. The limited number of animals reduces statistical power and restricts generalization of the findings.

Future studies should include larger sample sizes, dose optimization, and mechanistic investigations such as histological analysis of hair follicles and evaluation of hair growth phases (anagen/telogen ratio). Such studies would provide more robust evidence regarding the efficacy and mechanism of action of these plant‐based formulations.

Overall, the present findings suggest that the combination of *R*. *officinalis* and *R*. *communis* oils may have potential as a topical formulation for hair growth promotion under experimental conditions, warranting further investigation.

## 5. Study Limitations

This study has several important limitations that should be considered when interpreting the findings. First, the experiment was conducted as a preliminary in vivo screening study with a small sample size (*n* = 3 per group), which limits statistical power and increases the risk of Type II error. No formal sample size calculation or power analysis was performed, as the study was designed to generate exploratory biological data rather than to confirm efficacy. Second, although differences were observed in some treatment groups, the small sample size and inherent biological variability in animal models limit the robustness and generalizability of the findings. Third, the study was conducted in a murine model, and therefore the results cannot be directly extrapolated to human physiology or clinical outcomes, particularly in conditions such as alopecia, which involve complex immunological and hormonal mechanisms. Fourth, although macroscopic hair growth assessment and hair length measurements were used, more objective endpoints such as histological evaluation of hair follicles (such as anagen/telogen ratio) and molecular markers of hair growth were not performed, which limits mechanistic interpretation. Finally, potential variability in topical absorption and interanimal differences may have influenced the observed responses. Due to the small sample size and absence of histological or molecular endpoints, mechanistic conclusions remain speculative.

Despite these limitations, the study provides useful preliminary evidence supporting the biological activity of the tested plant extracts and highlights the need for further well‐powered, mechanistic, and eventually clinical studies to validate efficacy and safety.

## 6. Conclusion

The combination of *R*. *officinalis* and *R*. *communis* oils demonstrated a trend toward enhanced hair growth in a murine model under the conditions of this experiment. The combination showed improved performance compared with individual treatments and comparable trends with 2% minoxidil. However, these findings are preliminary and limited by the small sample size and preclinical nature of the study. Further studies involving larger sample sizes, detailed mechanistic evaluation, and clinical trials are required to confirm efficacy and safety. Overall, the results provide initial experimental evidence supporting the potential of this plant‐based combination for further investigation in hair growth research.

## Author Contributions

A.M., E.O., R.M., and F.O. were involved in the design of the study. A.M., R.M., and F.O. collected laboratory data. All authors were involved in the analysis of data. The first draft was done by E.O., and all authors were involved in the revision of the draft manuscript.

## Funding

No funding was received for this manuscript.

## Disclosure

All authors agreed to the final content.

## Conflicts of Interest

The authors declare no conflict of interest.

## Data Availability

The data that support the findings of this study are available from the corresponding author upon reasonable request.
